# Automatic Quality Assessment of Pork Belly via Deep Learning and Ultrasound Imaging

**DOI:** 10.3390/ani14152189

**Published:** 2024-07-27

**Authors:** Tianshuo Wang, Huan Yang, Chunlei Zhang, Xiaohuan Chao, Mingzheng Liu, Jiahao Chen, Shuhan Liu, Bo Zhou

**Affiliations:** College of Animal Science and Technology, Nanjing Agricultural University, Nanjing 210095, China; 2022805106@stu.njau.edu.cn (T.W.); 2021105038@stu.njau.edu.cn (H.Y.); 2020105039@stu.njau.edu.cn (C.Z.); 2021205020@stu.njau.edu.cn (X.C.); 2020205018@stu.njau.edu.cn (M.L.); chenjiahao@stu.njau.edu.cn (J.C.); 2022105002@stu.njau.edu.cn (S.L.)

**Keywords:** B-ultrasound imaging, deep learning, image classification, pork belly quality, real-time recognition

## Abstract

**Simple Summary:**

This study presents an automated intelligent technique for real-time identification and assessment of pork belly layers in B-ultrasound images. This non-invasive method can boost the efficiency of breeders in evaluating the layer count within pork belly. By integrating the imaging features of B-ultrasound with a deep learning architecture tailored for image classification, this approach delivers high-precision recognition and categorization of pork belly strata. The findings indicated that the deep learning model adeptly delineated the boundaries between adipose and lean tissues, precisely discerning various layer counts. The system was successfully implemented in a local setting and is now primed for practical deployment.

**Abstract:**

Pork belly, prized for its unique flavor and texture, is often overlooked in breeding programs that prioritize lean meat production. The quality of pork belly is determined by the number and distribution of muscle and fat layers. This study aimed to assess the number of pork belly layers using deep learning techniques. Initially, semantic segmentation was considered, but the intersection over union (IoU) scores for the segmented parts were below 70%, which is insufficient for practical application. Consequently, the focus shifted to image classification methods. Based on the number of fat and muscle layers, a dataset was categorized into three groups: three layers (n = 1811), five layers (n = 1294), and seven layers (n = 879). Drawing upon established model architectures, the initial model was refined for the task of learning and predicting layer traits from B-ultrasound images of pork belly. After a thorough evaluation of various performance metrics, the ResNet18 model emerged as the most effective, achieving a remarkable training set accuracy of 99.99% and a validation set accuracy of 96.22%, with corresponding loss values of 0.1478 and 0.1976. The robustness of the model was confirmed through three interpretable analysis methods, including grad-CAM, ensuring its reliability. Furthermore, the model was successfully deployed in a local setting to process B-ultrasound video frames in real time, consistently identifying the pork belly layer count with a confidence level exceeding 70%. By employing a scoring system with 100 points as the threshold, the number of pork belly layers in vivo was categorized into superior and inferior grades. This innovative system offers immediate decision-making support for breeding determinations and presents a highly efficient and precise method for assessment of pork belly layers.

## 1. Introduction

Pork belly, celebrated for its distinctive structure and flavor, is a culinary staple across diverse global cuisines [1]. It is rich in essential fatty acids and a certain amount of protein, providing a variety of nutrients for the human body [2]. The modern pork industry often aims to increase the lean meat content of pigs. However, this approach is counter to enhancing the quality of pork bellies, as the fat content significantly affects the quality characteristics of pancetta [3]. Additionally, the quality of pork belly is influenced by various factors, including genetics, rearing conditions, and sex, which can lead to a wide variation in the ratio of fat to lean meat layers [4]. Although the pork industry is currently trending towards enhancing the lean meat rate, the market’s appetite for pork belly persists, driven by its distinctive taste and nutritional benefits [5]. Genetic enhancement is a viable strategy for improving pork belly quality. Research indicates that the traits of pork belly and its components possess moderate to low heritability, suggesting that there is potential for quality improvement through genetic selection [6].

Accurate assessment of pork belly can help with better market segmentation. Traditional meat quality assessment methods, such as the “finger method”, are highly dependent on personal experience and tend to be more subjective [7]. Modern intelligent analysis technologies offer innovative solutions, such as deep Raman spectral analysis [8], near-infrared reflectance spectroscopy [9], and multivariate analysis [10]. These techniques facilitate real-time monitoring of fat content, rapid and precise detection and quantification of various meat components in pork, and even the prediction of pork belly tenderness. Ultrasound imaging technology stands out as a non-invasive and efficient tool for meat quality assessment [11]. It has demonstrated significant potential in the agricultural sector, particularly in livestock breeding [12]. This technology allows for real-time observation of muscle traits in pigs, minimizing harm to the animals and enhancing breeding efficiency.

In the 1990s, the automatic fat measurement system known as AutoFOM was introduced, employing ultrasound technology to assess parameters such as the lean meat percentage (LMP) and the fat thickness in the abdominal region of carcasses [13]. While the implementation of this system enhanced the efficiency of the measurement process, discrepancies in the evaluation protocols necessitate enhancements in accuracy. Furthermore, the full potential of B-ultrasound imaging has yet to be realized. Current obstacles in the field of ultrasound imaging are multifaceted, encompassing issues such as image noise [14] and data scarcity. This is particularly evident in the livestock sector, where advancements in deep learning research pertaining to ultrasound images have lagged behind. This area demands more extensive investigation and refinement to fully harness the capabilities of deep learning in analyzing ultrasound data.

Deep learning, a subset of machine learning, has found success across various domains by autonomously extracting intricate features from images. Convolutional neural networks (CNNs), in particular, are a potent class of neural networks specifically crafted for the analysis of image data, and they have become a dominant force in the realm of computer vision [15]. CNNs minimize the number of parameters needed through parameter sharing, which streamlines the training process and bolsters the network’s capacity for generalization. Applications of CNNs span semantic segmentation, image recognition, image classification, and natural language processing, among others. In the medical sector, image classification has consistently yielded high-precision outcomes. For instance, researchers proposed a medical application leveraging CNN methodologies and image quality assessment (IQA) algorithms for the classification of breast cancer [16]. Additionally, Li and colleagues developed a tailored CNN with shallow convolutional layers to classify interstitial lung disease (ILD) [17].

The application of deep learning technology in agriculture is growing in significance. It aids in constructing models that extract valuable insights from agricultural data, facilitates the automated performance of various farming tasks, and enhances overall productivity [18]. Within the realm of animal husbandry, deep learning is predominantly utilized for analyzing animal imagery and physiological metrics, detecting early signs of illness, forecasting disease outbreaks, observing behavioral patterns, and evaluating the health of animals [19]. Moreover, deep learning can be employed for trait measurement, which expedites the breeding process. Accurate and swift identification of pig body size is pivotal during the breeding phase, as it is instrumental for gauging their growth and developmental progress [20].

Therefore, this study explores the potential application of deep learning technology in the assessment of pork belly layers. We developed a deep-learning-based system that is capable of automatically extracting features from B-ultrasound images of pork belly, thereby facilitating an accurate evaluation of its quality and grading. The primary objective of this research was to devise a non-invasive and efficient method for real-time assessment of pork belly. By leveraging deep learning algorithms on B-ultrasound images, we aimed to bolster decision-making in breeding and enhance the control over meat quality. This innovative approach promises to revolutionize the pork industry by providing a reliable and swift means of quality assessment, thereby ensuring the production of high-quality pork belly that meets consumer expectations and industry standards.

## 2. Materials and Methods

### 2.1. Creation of the Dataset

The B-ultrasound image dataset was sourced from B-ultrasound scans of 160 pigs at local markets in Nanjing, where the pork belly of each pig was measured. Additionally, we also collected B-ultrasound image data from 58 fattened pigs, each aged six months, at Xuzhou Runwo Animal Husbandry Co., Ltd. in Jiangsu Province. The B-ultrasound imaging was performed using a MyLabTouch (Esaote, Genova, Italy), a high-definition veterinary B-ultrasound device manufactured by the Italian company Esaote (Genova, Italy). The device’s linear probe was configured with a detection depth of 15 cm and an operating frequency of 3.6 MHz.

The measurement method involved placing the B-ultrasound probe at three locations on the lower quarter of the abdomen (anteriorly between the 6th and 7th ribs, centrally between the last 3rd and 4th ribs, and posteriorly at the location of the 5th lumbar vertebra) to perform ultrasound measurements on live pigs and save the images. Out of the dynamic images obtained, three clear, correctly positioned, and measurable B-ultrasound images were selected and saved. After the measurement, images with distortion, blurriness, noise, or artifacts due to improper operation were screened out, and high-quality images with clear and distinguishable muscle and fat layer boundaries, uniform muscle texture, and no obvious shadows or artifacts were retained. The original instrument-saved images were cropped using the ROI (region of interest) function, and the image segmentation was manually completed in batches using the ImageTool website.

For pork belly, irrespective of the number of layers, the outermost layer is always the skin, followed by a fat layer, and the innermost is the muscle layer (excluding the slab of fat in the abdominal cavity). Consequently, the layer count of pork belly is typically odd. Reflecting this, images were sorted into three distinct categories: three-layer, five-layer, and seven-layer, and organized into corresponding folders. Illustrative examples for each category are presented in Table 1. The dataset comprised a total of 3984 images, which were partitioned into training and testing sets with an 8:2 ratio. Classification was determined by the number of distinct white lines observable at the interface between muscle and fat tissues. Typically, tissues with denser collagen content reflect ultrasound waves more effectively, resulting in whiter pixels in the corresponding images. Conversely, structures or tissues lacking collagen or with uniform composition do not reflect the waves and thus appear black in ultrasound imagery.

To foster diversity within the experimental dataset, the images were cropped to varying sizes, with dimensions spanning from 100 to 800 pixels. A visual representation illustrates the distribution of image sizes, where each point corresponds to an image of a particular size. The intensity of the point color indicates the prevalence of images at that size; darker shades signify a higher concentration of images, while lighter shades denote fewer images. Notably, the size of 200 × 200 pixels was the most densely represented. Overall, having a variety of sizes can reduce the impact on model training of the use of a single image size. In addition to this, data augmentation was performed on the dataset, including operations such as cropping, flipping, converting to tensor format, normalization, and standardization. Resizing was carried out to 256, and the cropping size was set to 224. The purpose of this approach was to adapt to the data size required for model input and to unify the format, reducing the interference in results caused by different settings and also enriching the diversity of the data. The code library used in this process was PIL (Python Image Library), a third-party image processing library for Python.

### 2.2. Model Selection

The selection of models for this study encompasses a diverse range, including ResNet18, ResNet50, ResNet101, ResNet152, VGG16, VGG19, and AlexNet. These models represent three distinct architectural series, each with a unique set of features and capabilities. A detailed comparison of the characteristics that distinguish these series is presented in Table 2.

To ensure the rigor and consistency of the experimental results, the seven models used the same dataset during the training process, with identical experimental equipment, configurations, and environments. The cloud server platform used was Featurize (Garlic Block, Chengdu, China), with the hardware equipment being NVIDIA RTX3060, featuring 12 GB of video memory, 5 × E5-2680 v4 CPUs, 26 GB of RAM, and a 350 GB hard disk. The parameters of the toolkit used are shown in Table 3. Since the dataset used in this study did not intersect with the original dataset used for transfer learning [24], it was necessary to randomly initialize the model weights and retrain all layers on our own dataset, to ensure the independence of training.

### 2.3. Model Evaluation Index

To assess the performance of the algorithmic model, the following metrics were utilized: accuracy, recall/true positive rate (TPR), specificity, precision, F1 score model, false positive rate (FPR), along with their respective macro and weighted averages [25]. These are illustrated in Equations (1)–(6) and Table 4.

(1) $$\mathrm{Accuracy}=\frac{\mathrm{TP}+\mathrm{TN}}{\mathrm{TP}+\mathrm{TN}+\mathrm{FP}+\mathrm{FN}}$$

(2) $$\mathrm{Recall}=\mathrm{TPR}=\frac{\mathrm{TP}}{\mathrm{TP}+\mathrm{FN}}$$

(3) $$Specificity=\frac{TN}{TN+FP}$$

(4) $$Precision=\frac{TP}{TP+FP}$$

(5) $$F1_{score}=2\times \frac{Precision\times Recall}{Precision+Recall}=\frac{2TP}{FP+FN+2TP}$$

(6) $$\mathrm{FPR}=1-\mathrm{Specificity}=\frac{FP}{FP+TN}$$

where TN and TP represent the counts of negative and positive instances or pixels, respectively, that were correctly identified. FN refers to positive instances that were incorrectly identified as negative, while FP refers to negative instances that were incorrectly identified as positive.

Accuracy was used to evaluate the proportion of instances in the pork belly fat B-ultrasound image category that were predicted as positive out of the total number of instances. Generally, the higher the accuracy, the better the classification algorithm. Recall, also known as the true positive rate (TPR), refers to the proportion of instances predicted as positive out of all actual positive instances, indicating the proportion of actual correct identifications. Specificity refers to the proportion of incorrectly predicted instances out of all actual negative instances. The F1 score is the harmonic mean of precision and recall, and the higher the F1 score, the more effective the test method. TPR is inversely related to FPR. The higher the TPR, the higher the probability of selecting the correct sample, and the lower the FPR. F1Score represents the value of the F1 score, which is the highest value of recall and precision. Macro avg. (macro average) directly averages the evaluation metrics for each class, that is, the precision, recall, and F1 score. Weighted avg (weighted average) calculates the average of the evaluation metrics weighted by the number of samples (support), taking into account the proportion of each class’s sample size in the total samples, which is an improvement over the macro average.

The precision–recall (PR) curve and the receiver operating characteristic (ROC) curve are often used to measure the performance of a model. The PR curve calculates the recall and precision of samples by reclassifying them after changing the threshold once, and then uses these as coordinate values to plot on a plane coordinate graph. The PR curve is more sensitive to samples and measures the classifier’s ability to classify in the face of imbalanced data. The receiver operating characteristic curve (ROC) is also known as a sensitivity curve, where each point on the curve reflects the same level of sensitivity. The ROC curve can be used for threshold selection and for comparing different models. The area under the ROC curve (AUC) [26] and the area under the PR curve (AP) are often used to evaluate the effectiveness of diagnostic models.

For enhancing interpretability, three methods were employed: Grad-CAM, integrated gradients, and occlusion sensitivity analysis. The model’s recognition capabilities could be visualized by randomly inputting images, which allowed us to identify the most important regions for model recognition and to assess the model’s reliability.

### 2.4. Local Deployment

The successful exploration of the aforementioned methods led to the next step, which was to deploy the optimized model results to bring the experiment to fruition. The evaluation of the quality of pork belly needs to be conducted in real-time at the breeding site, so that staff can make quick decisions based on the evaluation results. Offline embedded end deployment can meet this real-time requirement, ensuring that the evaluation results can be fed back immediately. A one-time investment in deploying local hardware and software resources can be used for a long time, thus reducing operational costs. At the same time, keeping data processing local reduces data transmission and helps protect sensitive information in breeding work.

The initial exploration of local deployment used ONNX Runtime (Microsoft, Redmond, WA, USA) as the inference engine [27], deploying the training results obtained in the previous chapter on the computer to achieve the recognition of the characteristics of the number of layers in pork belly in B-ultrasound images, monitoring videos, and even real-time probe images. It is hoped that further improvements can be made to directly embed the system into B-ultrasound equipment, allowing for real-time qualitative determination of traits during the monitoring process.

**Figure 1:**
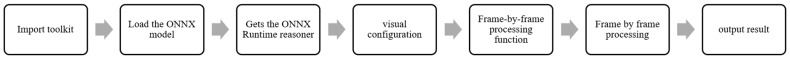
System design flowchart.

The system is capable of recognizing various data types, encompassing static images, video files, and live camera feeds. Static images are recognized and displayed in a straightforward manner. Video data, on the other hand, require a frame-by-frame processing approach. Once the individual frames have been predicted, they are reassembled into a coherent video sequence. For real-time camera feeds, the system captures images using the camera in real time, processes these images through the video data processing pipeline, and outputs results on the fly. The system’s design is visualized in the flowchart presented in Figure 1, which provides a schematic overview of the process flow and the interconnections between different components of the system.

#### 2.4.1. File Preparation

**Figure A1:**
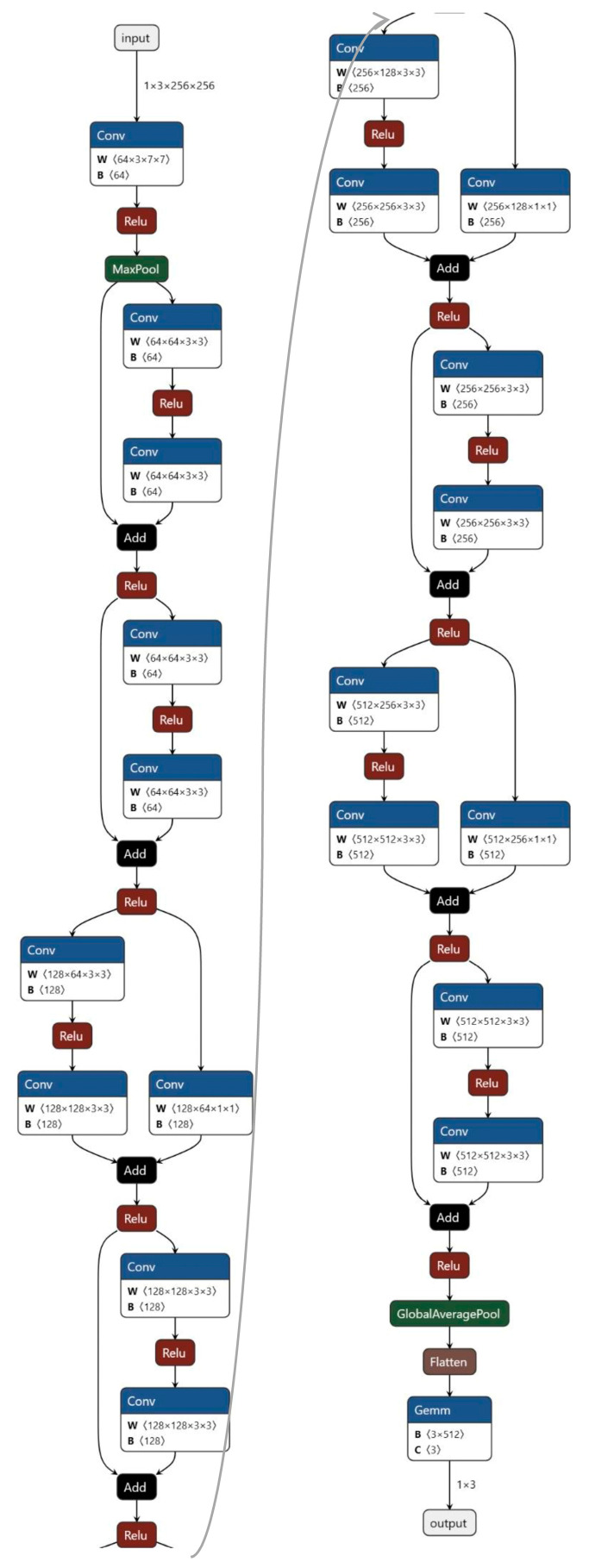
Structure diagram of the ONNX model.

The PyTorch file of the ResNet18 model retains the feature pattern with the highest accuracy after the 22nd iteration. The data format is transformed from PyTorch to ONNX using the ONNX export function, specifying the operator version as 11. At this point, the file name suffix becomes “onnx” and the completeness and correctness of the conversion are verified. The newly generated file is then dragged into the Netron platform (https://netron.app/, accessed on 8 April 2024) for visualization, as shown in Figure A1. The image displays information about the ONNX model, including the model version, as well as the names and data types of the model’s inputs and outputs. Each operator node also records specific information such as operator attributes, graph structure, and weights. The indices of the three categories of 3, 5, and 7 layers are marked as 0, 1, and 2, respectively, and saved as a CSV file. This is used for ID retrieval after each prediction, where the confidence level of each ID is assigned to the corresponding category and then outputted.

#### 2.4.2. Pretreatment

Model preparation starts by importing the toolkit and loading the prepared ONNX format model file into the ONNX runtime interpreter by running the InferenceSession function. This constructs the format of the input and output data according to the names previously given during file preparation. The steps of crop, flip, convert to tensor format, normalize, and standardize are performed for the predicted image. Except for cropping that requires changing the image size to 256, the rest of the parameters remain the same. The final image format is {1,3,256,256}.

#### 2.4.3. Inference Prediction

The images or video data to be predicted by the model are imported, as well as the CSV file containing the category indices. Inference is performed, the confidence levels for each category are sorted and matched with the category indices, and finally the results are printed out.

For video data analysis, the approach involves treating the video as a sequence of individual image frames. The model operates by first determining the total frame count of the video, which equates to the number of images it contains. It then proceeds to predicting each frame sequentially. Once all frames have been analyzed, they are compiled back into video format. In the case of real-time camera image data, the OpenCV-Python toolkit is employed. The VideoCapture function is utilized to access the system’s camera, capturing images for input purposes. The captured images in BGR format are transformed into an RGB format using Pillow, which is compatible with OpenCV’s requirements. The model performs real-time inference on a frame-by-frame basis. The results of these predictions are superimposed onto the upper right corner of each image. To monitor performance, the processing time for each image is recorded in a time format, and the system’s efficiency is quantified using the frames per second (fps) metric, providing a clear indication of how smoothly the real-time analysis is being conducted.

## 3. Results

### 3.1. Model Performance Comparison

Table 5 records the number of iterations and the corresponding accuracy and loss parameters when each model achieved its highest level during the training process. It can be seen from the table that all seven models were capable of recognizing different layers in belly fat B-ultrasound images at a high level in terms of accuracy and loss function performance. Compared with the VGG and AlexNet models, the ResNet series of models had lower loss values, indicating better performance on the training data. Among them, the best-performing model was ResNet18, with an accuracy of 0.99999 and a loss of 0.14785.

Table 6 presents the performance of each model on the test set. ResNet18 also demonstrated good performance, with a high accuracy of 0.96226, precision of 0.96095, recall of 0.95576, and F1 score of 0.95753, despite having fewer training epochs. ResNet152 showed a higher loss after 30 epochs, but the accuracy remained high, which may indicate that the model had some degree of overfitting. ResNet50 had a lower loss and higher performance metrics with fewer epochs, showing a good balance of efficiency and performance. VGG19 and VGG16, although not trained for many epochs, had slightly lower performance metrics than the ResNet series models, especially in terms of loss. AlexNet had the lowest performance metrics among all models, particularly in accuracy and F1 score, which may have been due to its relatively older and fewer layers in the network structure.

**Figure 2:**
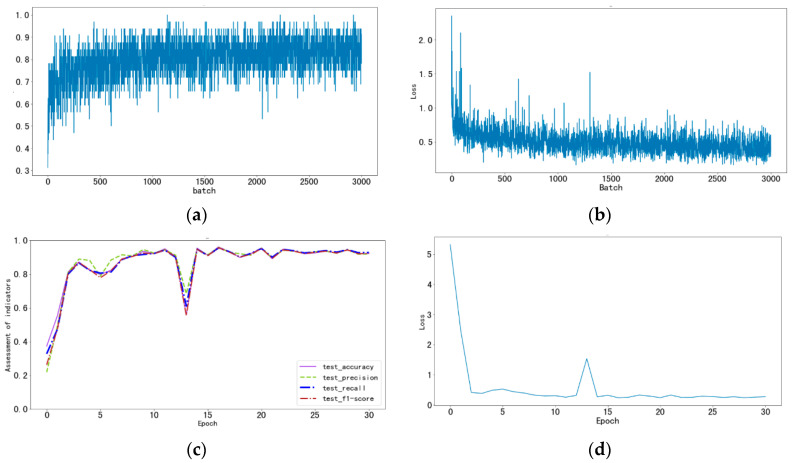
The variation curves of the metrics. (**a**) Variation curve of the accuracy of the training set; (**b**) variation curve of the loss function of the training set; (**c**) variation curve of each evaluation index of the test set; (**d**) variation curve of the loss function of the test set.

The variation curves of the various metrics for the ResNet18 model on both the training and test sets are shown in the Figure 2. The accuracy on the training set increased rapidly, reaching its peak after approximately 2500 batches and stabilizing around 0.85, suggesting the model may have been nearing its performance plateau. The loss function initially decreased quickly, with fluctuations later on, but overall trended downward and stabilized. During training, the model exhibited stability despite minor fluctuations. Evaluation on the test set showed that all metrics rose swiftly in the initial epochs, dipped around the 5th epoch, possibly due to a high learning rate or challenging data batches, and then rebounded and continued to improve, indicating parameter adjustments or enhanced data understanding. After about 10 epochs, the metrics stabilized at a higher level, with accuracy, precision, recall, and F1 score maintaining close proximity, demonstrating a balanced performance across metrics. Although there was a slight decline in the final epochs, the overall performance did not degrade significantly. Similarly, the loss function graph showed corresponding effects, with a faster overall downward trend and stability.

Considering the performance of the seven models on both the training set and the test set, ResNet 18 was ultimately chosen as the best model for subsequent deployment. Table 7 represents the recognition of the categories of 3-layer, 5-layer, and 7-layer belly fat B-ultrasound images, as well as their weighted and macro averages. For the classification of the 3-layer images, the model exhibited extremely high performance with the best metrics, indicating that the model made almost no mistakes when distinguishing between the three categories. For the classification of the 7-layer images, these metrics were lower than those of the 3-layer and 5-layer images, but still remained above 0.91, showing that the model maintained a high performance when dealing with more complex tasks. The macro average here was slightly lower than the weighted average, suggesting that the model’s performance was slightly lower on categories with fewer samples.

**Figure 3:**
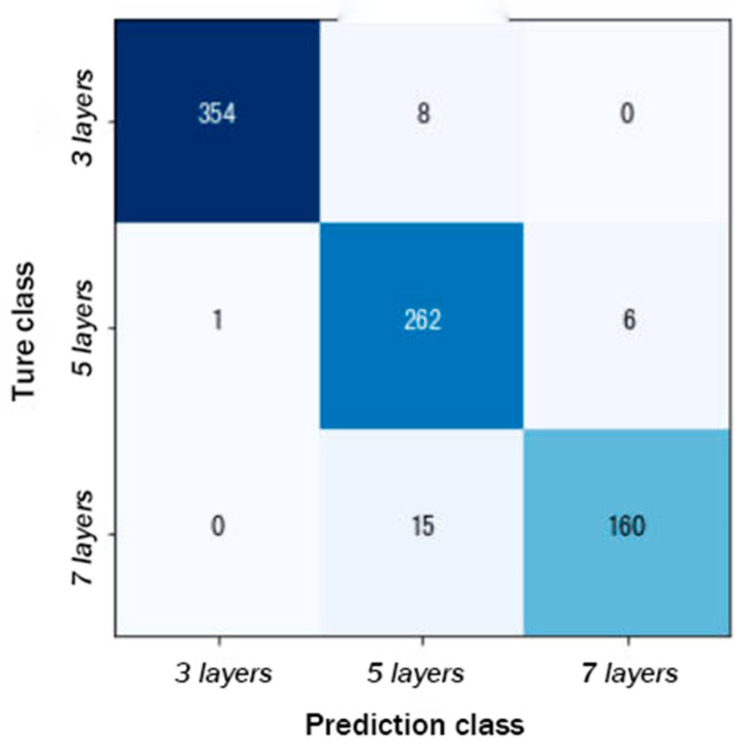
Confusion matrix. The number on the main diagonal represents the number predicted correctly. The darker the color, the greater the number.

Figure 3 presents a confusion matrix. The elements on the diagonal represent the number of samples correctly classified by the model. For the categories of 3 layers, 5 layers, and 7 layers, the model correctly classified 354, 262, and 160 samples, respectively. The off-diagonal elements represent the instances of misclassification. The model tended to incorrectly classify samples as the 7-layer category (15 samples) rather than the 3-layer category (1 sample). This may suggest that the model encountered some difficulties in differentiating between the 5-layer and 7-layer categories. The reason for this could be that these two categories are more similar in their features, or the model’s ability to distinguish the features of these two categories was insufficient.

### 3.2. Model Performance Evaluation

**Figure 4:**
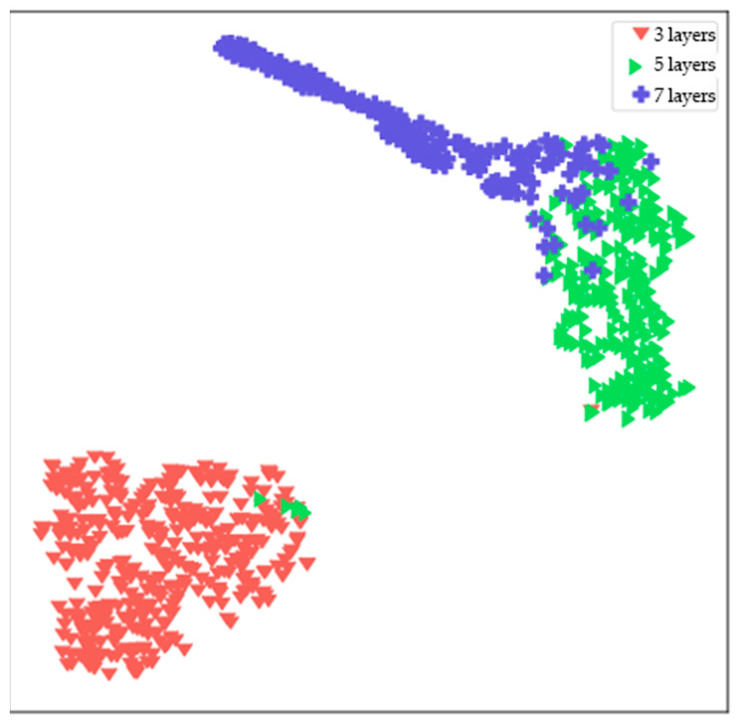
Scatter plot of t-distributed stochastic neighbor embedding.

To further clarify the analysis of the model’s clustering situation. Through t-SNE (t-distributed stochastic neighbor embedding) semantic dimensionality reduction visualization technology [28], the last ReLU layer of the network was mapped onto a 2D space as a feature embedding map to check the degree of distinction between the three categories of the model, with the results shown in the Figure 4. Each point in the figure represents an image in the test set, with each category represented by a different color. The 3-layer category forms a relatively tight cluster and is clearly separated from the other categories. This indicates that the data features learned by the model for the 3-layer category were relatively concentrated. There is some overlap between the data points of the 5-layer and 7-layer categories in the space, especially in the central area. It is possible that there are similarities in some dimensions, causing them to be closer to each other in the space after dimensionality reduction.

**Figure 5:**
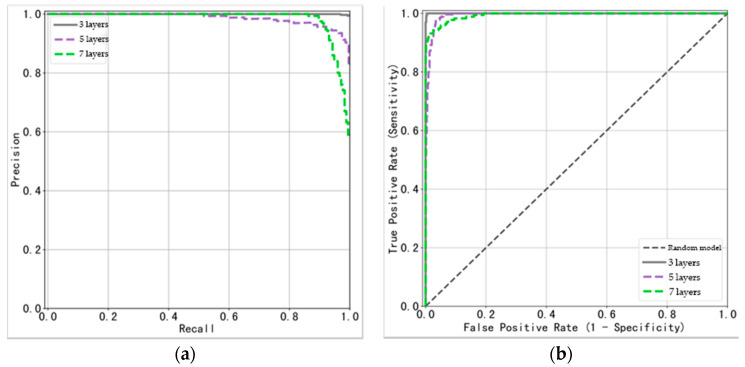
Model performance evaluation: (**a**) ROC curve; (**b**) P–R curve.

Figure 5a presents the P–R (precision–recall) curves for the classification performance of each category of the model. The ideal state of model performance is when the precision remains at a high level as the recall rate increases, meaning the closer the curve is to the upper right corner of the image, the better the classification performance of the model. The green line represents the P–R curve for the 3-layer category, which is almost a perfect square, indicating that the model achieved very good performance in the classification of this category. The P–R curves for the 5-layer and 7-layer categories are lower compared to the 3-layer category, especially for the 7-layer category, whose P–R curve shows a more pronounced decrease in precision as the recall rate increases.

The ROC (receiver operating characteristic) curves for the three categories are shown in Figure 5b. Compared to the P–R curves, which focus more on the proportion of positive classes, the ROC curves take into account both positive and negative classes. There is a one-to-one correspondence between the P–R curves and the ROC curves. The more the curve tends towards the upper left corner, the better the performance of the model. The curves in the figure also further demonstrate the excellent performance of the ResNet18 model.

The detailed quantitative results are outlined in Table 8. For the three classifications of pork belly ultrasound images—3-layer, 5-layer, and 7-layer—the average precision (AP) and area under the curve (AUC) values were remarkably close to 1. This proximity underscores the robust classification performance of the ResNet18 model. An AP or AUC value nearing 1 signifies that the model maintains a high true positive rate while keeping the false positive rate low, across various threshold settings. Such a characteristic is highly advantageous for a classification model, as it indicates a reliable ability to accurately classify images. The consistently high AP and AUC values observed across all three categories provide evidence that the model exhibited uniform excellence in its predictive capabilities. This demonstrates the model’s proficiency in discerning among the distinct classes of pork belly ultrasound images, reinforcing the validity of the ResNet18 model as an effective tool for this application.

### 3.3. Interpretability Analysis

To understand the feature patterns learned by deep learning models, interpretability analysis is commonly used to visualize the features that contribute most to the model’s predictions, explain the motifs and patterns recognized by the network, and provide guiding ideas for subsequent tissue identification.

**Figure 6:**
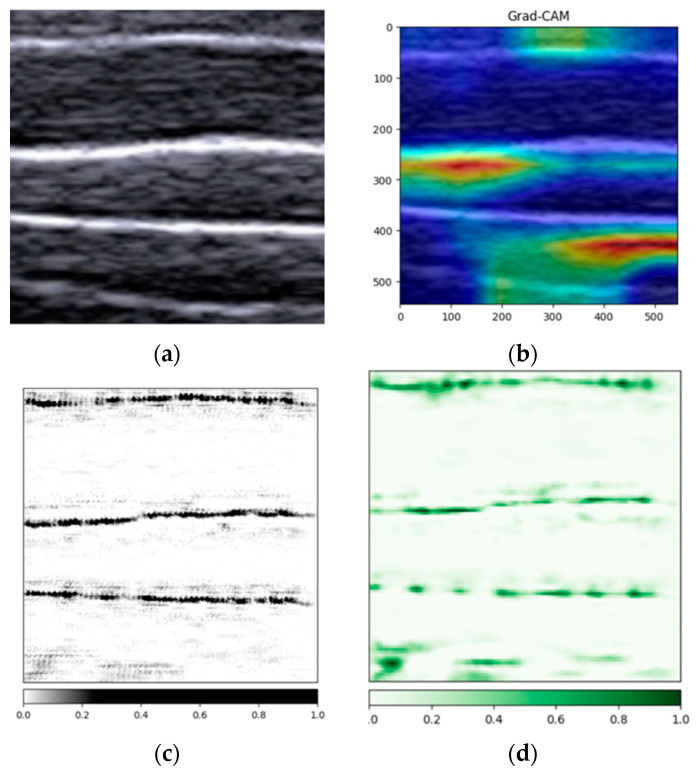
Visualization results of three types of interpretability analysis: (**a**) original image; (**b**) grad-CAM method. The color in the heat map indicates how much the pixel contributes to the prediction result; (**c**) integrated gradients method. Darker colors indicate that the region has a greater impact on the model’s predictions; (**d**) occlusion interpretability analysis method. Darker blue colors indicate that the region has a greater impact on the model’s predictions.

Figure 6 presents an array of interpretable analysis techniques used to elucidate the decision-making process of deep learning models. Figure 6a displays an original B-ultrasound image of pork belly layers, which acts as a reference point for evaluating the effectiveness of various interpretability methods. Figure 6b illustrates the grad-CAM method [29]. This method generated a heatmap with darker red regions that correspond to the boundaries between fat and lean layers, although not all boundary areas are covered. The heatmap’s irregular spot-like patterns may account for the areas not fully aligned with human perception of the boundaries. Moving to Figure 6c, we can observe the integrated gradient algorithm [30] at work. The algorithm’s output highlights pixel areas that match well with the bright white boundaries of the fat and lean layers in the original image. However, the clarity of the last feature at the bottom is not as distinct, indicating a weaker capture effect by the algorithm in that region. Lastly, Figure 6d showcases the occlusion interpretability analysis method [31]. This technique involves using a small slider to traverse the boundary area, allowing for a detailed analysis of the corresponding features. It offers a higher degree of precision compared to the previous methods and is adept at highlighting finer details.

By integrating the three aforementioned interpretive methods—grad-CAM, integrated gradients, and occlusion analysis—the characteristics of pork belly ultrasound images discerned by the ResNet18 model aligned with human recognition patterns. This consistency validated the model’s accuracy and reliability, suggesting that it is well-suited for deployment on terminal devices for practical applications.

### 3.4. Interpretability Analysis

#### 3.4.1. Single Image Prediction

Establishing a prediction function tailored for single images is essential, particularly given that the majority of measurement devices operate with limited configurations. In the context of veterinary practice, once an area of interest on a pig has been scanned using a standard B-ultrasound device, the image obtained can be paused, or “frozen”, at the optimal moment. This frozen image is then saved and subsequently used for detailed measurement and analysis of the trait data it contains. This approach ensures that even with lower-end equipment, valuable B-ultrasound images can be captured and later analyzed to extract meaningful insights into the characteristics of pork belly. The prediction function, therefore, plays a critical role in the workflow, allowing for the efficient translation of raw ultrasound data into actionable information that can inform decisions in the pork industry.

To utilize the prediction function, one must first select the image intended for prediction. Upon clicking the run command, the system processes the image and outputs both the recognized category and the associated confidence level. Three randomly selected images from each category were input into the model. The output results are shown in Table 9. The category with the first confidence level matches the actual category of the image, proving the strong generalization ability of the model. This demonstrates that the model can provide effective predictions on data it has not encountered during training. Furthermore, the confidence level reported by the system is directly related to the quality of the input image, suggesting that higher quality images are likely to yield higher confidence scores in the predictions.

#### 3.4.2. Video and Camera Real-Time Picture Prediction

**Figure 7:**
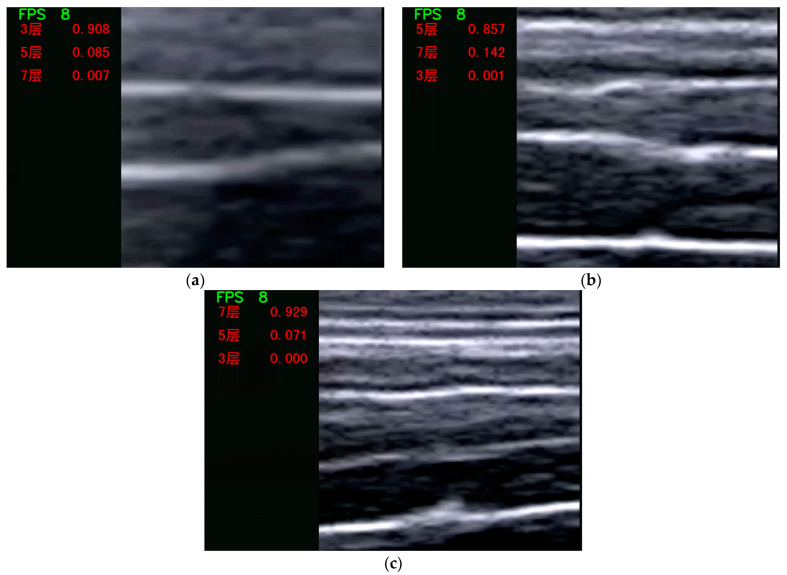
Real-time video or camera output (**a**) 3 layers; (**b**) 5 layers; (**c**) 7 layers.

Ultrasound instruments capture images of tissue morphology by emitting ultrasonic waves through a probe, which are then visualized on a screen. The input mechanism of the prediction system can be seamlessly integrated with the probe, enabling real-time identification and immediate display of prediction outcomes during the scanning process. For added convenience, the measurement procedure can be recorded in video format and subsequently fed into the system, ensuring that the results produced by both methods remain consistent. Figure 7 illustrates a test scenario where this function was executed on a local computer, utilizing the computer’s camera to process the images. Given the performance constraints of the computer in question, the frame rate (FPS) hovered around 8–9 frames per second. Despite this limitation, the output prediction’s confidence level was still capable of exceeding 70%. The overall prediction accuracy was highest for the three-layer category. However, due to the original training of the model, there may occasionally be instances where the confidence levels for the five-layer and seven-layer categories are similarly high.

### 3.5. Quantitative Assessment of the Number of Layers of Pork Belly In Vivo in Pig Breeding

To achieve an objective and direct assessment of the number of layers of pork belly in vivo, a systematic approach was employed, which involved three B-ultrasound images from the anterior, mid-abdominal, and posterior portions of the pig abdomen for prediction purposes. The prediction outcomes were then quantified using a scoring system. The scoring was calculated using the formula provided:

(7) $$\mathrm{Score}=50C_{3}+100C_{5}+150C_{7}$$

where *C*_3_ represents the confidence level associated with the prediction for the three-layer category, *C*_5_ represents the confidence level for the five-layer category, and *C*_7_ represents the confidence level for the seven-layer category. The numerical weights (50, 100, and 150) assigned to each confidence level reflect the perceived quality or desirability of each category, with higher numbers of layers potentially indicating higher quality. This scoring system serves as a grading tool for the pork belly, allowing for a standardized assessment. It translates the model’s predictions into a quantifiable score that can be easily interpreted and compared. The higher the score, the higher the quality of the pork belly, providing a clear and direct measure for quality assessment. This approach allows for a more precise and data-driven evaluation of pork belly quality, which can be particularly useful in the pork industry for making informed decisions regarding breeding, processing, and marketing. An example of quantitative assessment of pork belly is shown in Table 10.

## 4. Discussion

This study leveraged a range of established image classification models to classify and identify the muscle and fat boundary line characteristics in porcine abdominal fat B-ultrasound images. Following customized parameter tuning and a comprehensive evaluation of various performance metrics, the optimal ResNet18 model was chosen. Further analysis, including visualization results such as the confusion matrix, t-SNE distribution, P–R (precision–recall) curves, and ROC (receiver operating characteristic) curves, as well as interpretability analysis, revealed that the model’s identified feature locations aligned with human-annotated judgment points. This concurrence suggested that the model’s learning outcomes were trustworthy and positions it as a suitable baseline for future model deployment endeavors. Moreover, an initial implementation for recognizing the fat and lean layer traits in B-ultrasound images of pig belly fat was conducted on a local computer terminal. The system is designed to accommodate three types of inputs: static images, video files, and live camera streams, ensuring flexibility to cater to diverse device capabilities.

In the preliminary phase of the research, the precision capabilities of semantic segmentation technology were thoroughly examined, with initial experiments conducted to employ this technique. A variety of models were tested to delineate the boundary between adipose and lean tissues within porcine abdominal fat B-ultrasound images. Despite several tests, the accuracy achieved by metrics such as IoU was less than 70 and did not meet the standards for industrial applications. Consequently, the strategy pivoted towards utilizing image classification technology. This new approach focused on classifying images based on the count of fat and muscle tissue layers, fulfilling the recognition objective. This method also bypasses the labor-intensive process of manual image annotation, simplifying the requirement to classification by layer count. Subsequently, the model was trained to discern regions within the images that exerted a substantial influence on the classification outcome. Through this learning process, the model explores and captures critical features and identifies underlying patterns that are instrumental in distinguishing between different layer configurations.

We investigated the application of transfer learning, capitalizing on the training knowledge from established models to address the classification of pig belly fat images. Despite the absence of the specific images used in this experiment from the previous dataset, the underlying knowledge patterns were leveraged to enhance the initial model. This approach circumvented the need for training from the ground up, significantly conserving both time and resources. In the process, multiple models were evaluated, and the superior ones were chosen for further analysis. We delved into identifying the regions from which the models extracted features, visualizing the learning process to facilitate subsequent technical refinements.

At present, the output for the category with the highest confidence level typically stabilizes at approximately 70%. While there may occasionally be instances where the confidence levels for the 5-layer and 7-layer categories are similar, with confidence being allocated to non-primary layers, and this could be influenced by factors such as image noise, indistinct tissue layer boundaries, or the use of small cropping sizes. It is important to note that confidence level is not the sole metric for prediction accuracy. It must be assessed holistically alongside other indicators, including accuracy and recall rate, as discussed in the model validation in the preceding chapter [32].

The pivotal factor in determining the output results was the predictive model’s overall performance during its initial training phase. Although the transition from the PyTorch format to the ONNX format enhanced the efficiency and minimized memory usage through various technological advancements, discrepancies between formats can potentially lead to a degradation in accuracy and an escalation in model complexity. This necessitates further optimization in configuration settings. Beyond software performance [33], the computational and display capabilities of the B-ultrasound device also play a crucial role in the system’s efficiency. The model’s operation consumes a portion of the CPU and GPU memory, as well as other computer resources [34]. It is essential to verify that the device’s terminal model is compatible with the required version of the toolkit for operation. Once the software and hardware issues impacting the system’s overall performance have been addressed, future work can focus on expanding the standard dataset and refining the model’s recognition capabilities. Subsequently, the model will be encapsulated into an intuitive system front end designed for user-friendly operation. The ultimate goal is to seamlessly integrate the system with B-ultrasound equipment, thereby enhancing its practicality and contributing to the advancement of intelligent breeding practices.

This research presents an innovative approach to evaluating pork belly quality through deep learning analysis of B-ultrasound images, offering real-time, high-accuracy decision support. Implementing this system at breeding facilities has the potential to markedly enhance both the efficiency and precision of meat quality assessments. Nevertheless, this study had some limitations due to the inadequate size and diversity of the sample dataset. For example, the imbalance between model training data may have affected the training results of the model, because there were more samples of pork belly with fewer layers. To further improve this study, future efforts should be concentrated on expanding the dataset to encompass more breeds, B-ultrasound images of pigs at different ages, exploring other deep learning frameworks, and integrating a broader range of meat quality metrics to make the system more comprehensive.

## 5. Conclusions

This study successfully harnessed the power of deep learning for the real-time assessment of pork belly quality through the analysis of B-ultrasound images. By employing an image classification algorithm, we were able to identify and classify the fat and lean layers present in ultrasound images of pork. The ResNet18 model demonstrated exceptional accuracy, emerging as a dependable instrument for breeders to make well-informed decisions. Visualizations through t-SNE, along with ROC curves and other evaluative metrics, illustrated that the model’s learned recognition areas were in good alignment with the assessments made by human experts. Additionally, the ONNX Runtime inference engine was effectively utilized to establish a preliminary local deployment system. This system was adept at recognizing the fat and lean layer counts in B-ultrasound images of pork belly. The system’s versatility was evident in its ability to process diverse data inputs, including static images, video files, and live camera feeds. It consistently delivered output results with a confidence level exceeding 70%. Drawing from the experimental outcomes, this study proposed a computational method for the quality grading of pork belly, which stands to benefit the pork industry by enhancing the objectivity and efficiency of quality evaluations.

## Figures and Tables

**Table 1:** Number of samples in each train and test set.

Class	Train Set	Test Set	Total
3 layers	1449	362	1811
5 layers	1036	258	1294
7 layers	704	175	879

**Table 2:** Advantages of the models and reasons for their selection.

Model	ResNet [21]	VGG [22]	AlexNet [23]
Advantageous field	Image recognition and performance do not degrade with increasing depth	Visual recognition tasks such as image recognition and image classification	Image recognition, classification
Reasons for choice	Deep structure and residual connections enable it to learn deeper, discriminative features suitable for processing such images	The ability to capture detail allows it to identify subtle differences in muscle and fat layers	An early breakthrough in deep learning, improved training speed and reduced overfitting

**Table 3:** Toolkits and their versions.

Name	Version
Python	3.7
PyTorch	1.1
CUDA	11.3
GCC	9.3
mmseg	1.1.1
mmcv	2.0.0
opencv-python	4.5.4.60
pillow	8.4.0
matplotlib	3.5.0
mmdet	3.2.0

**Table 4:** Definition of averages.

	Precision	Recall	F1	Accuracy
weighted avg.	$\frac{\sum \left(n_{i}\times P_{i}\right)}{\sum n_{i}}$	$\frac{\sum \left(n_{i}\times R_{i}\right)}{\sum n_{i}}$	$\frac{\sum \left(n_{i}\times F1_{i}\right)}{\sum n_{i}}$	$\frac{\sum \left(n_{i}\times A_{i}\right)}{\sum n_{i}}$
macro avg.	$\frac{1}{n}\sum_{i=1}^{n}P_{i}$	$\frac{1}{n}\sum_{i=1}^{n}R_{i}$	$\frac{1}{n}\sum_{i=1}^{n}F1_{i}$	$\frac{1}{n}\sum_{i=1}^{n}A_{i}$

**Table 5:** Performance indicators of the overall classification of each model on the training set.

Model	Epoch	Loss	Accuracy
ResNet18	25	0.14785	0.99999
ResNet50	12	0.15995	0.96875
ResNet101	28	0.17524	0.96875
ResNet152	27	0.14464	0.96875
VGG16	17	0.20045	0.96875
VGG19	25	0.18962	0.96875
AlexNet	29	0.20778	0.93750

**Table 6:** Performance indicators of the overall classification of each model on the test set.

Model	Epoch	Loss	Accuracy	Precision	Recall	F1-Score
ResNet18	22	0.19767	0.96226	0.96095	0.95576	0.95753
ResNet152	30	0.22695	0.94665	0.95033	0.94567	0.94594
ResNet50	16	0.23123	0.96154	0.95817	0.95513	0.95625
VGG19	20	0.24573	0.93797	0.93262	0.92786	0.92941
ResNet101	15	0.25776	0.95037	0.95153	0.93828	0.94271
VGG16	9	0.30223	0.94789	0.95038	0.93323	0.94003
AlexNet	30	0.34298	0.90199	0.89556	0.89794	0.89479

**Table 7:** Evaluation indicators for the classification of each category in the model test set.

	Precision	Recall	F1-Score	Support	Accuracy
3 layers	0.99718	0.97790	0.98745	362	0.97790
5 layers	0.91930	0.97398	0.94585	269	0.97398
7 layers	0.96386	0.91429	0.93842	175	0.91429
Macro avg.	0.96011	0.95539	0.95724	806	0.95539
Weighted avg.	0.96395	0.96278	0.96292	806	0.96278

**Table 8:** Quantitative indicators of performance curves for each category.

Class	AP	AUC
3 layers	0.99976	0.99980
5 layers	0.97716	0.99043
7 layers	0.97846	0.99159
macro avg.	0.98513	0.99394
weighted avg.	0.98774	0.99495

**Table 9:** Images and their output results.

Class	Images	Output Results
3 layers	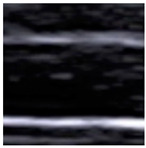	3 layers: 75.335 5 layers: 22.778 7 layers: 1.8870
5 layers	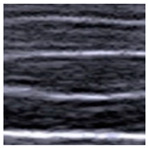	5 layers: 88.954 7 layers: 11.002 3 layers: 0.0430
7 layers	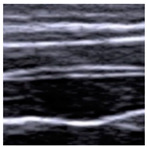	7 layers: 61.612 5 layers: 38.360 3 layers: 0.0280

**Table 10:** Quantitative assessment of pork belly layers in pig in vivo determination **^1^**.

Image	Predicted Class	Confidence	Scores	Partial Scores	Total Score
703310_anterior.jpg	5 layers	0.5914	59.14	119.25	121.19
	7 layers	0.3968	59.52		
	3 layers	0.0118	0.59		
703310_mid-abdominal.jpg	7 layers	0.9194	137.91	145.855	
	5 layers	0.0783	7.83		
	3 layers	0.0023	0.115		
703310_posterior.jpg	5 layers	0.828	82.8	98.475	
	3 layers	0.1011	5.055		
	7 layers	0.0708	10.62		

^1^ During the in vivo determination of backfat thickness and eye muscle area, the belly of the abdominal ribs of the pig can be measured on the anterior, mid-abdominal, and posterior portions of the abdomen using a B-ultrasound instrument at the same time. According to the method in Equation (7), the quantitative assessment of pork belly was predicted to represent the belly meat quality score of the pig. This makes the method more convenient to apply in breeding.

## Data Availability

Data are contained within the article: The original contributions presented in the study are included in the article, further inquiries can be directed to the corresponding author.
